# The Electoral Consequences of Affective Polarization? Negative Voting in the 2020 US Presidential Election

**DOI:** 10.1177/1532673X221074633

**Published:** 2022-01-27

**Authors:** Diego Garzia, Frederico Ferreira da Silva

**Affiliations:** 1 30656 University of Lausanne Faculty of Social and Political Science, Lausanne, Switzerland

**Keywords:** affective polarization, negative personalization, negative partisanship, political behavior, retrospective voting

## Abstract

About one third of American voters cast a vote more “against” than “for” a candidate in the 2020 Presidential election. This pattern, designated by negative voting, has been initially understood by rational choice scholarship as a product of cognitive dissonance and/or retrospective evaluations. This article revisits this concept through the affective polarization framework in the light of the rise of political sectarianism in American society. Based on an original CAWI survey fielded after the 2020 election, our regression analysis demonstrates that the predicted probability of casting a negative vote significantly increases among individuals for whom out-candidate hate outweighs in-candidate love. Negative voting is less prevalent among partisans as their higher levels of in-group affection can offset out-group contempt. By asserting the enduring relevance of negative voting in American presidential elections, we aim at stimulating further research and discussion of its implications for democratic representation.

## Introduction

Not unlike the most recent presidential contests, also the 2020 US Presidential election was characterized by widespread negativity. This occurrence fits with a theoretical account of rising *political sectarianism* among American voters, whereby out-party hate has progressively emerged as a stronger force than in-party love (Finkel et al., 2020).

A growing body of research has documented an intensifying affective polarization trend among the US electorate (Abramowitz & Webster, 2016; Boxell et al., 2020; Iyengar et al., 2012). However, much less is known about the political and electoral consequences of affective polarization, “as most studies have focused on the more surprising apolitical ramifications” (Iyengar et al., 2019: 139). In this paper, we contribute to filling this gap by revisiting the notion of *negative voting*, that is, an electoral choice more strongly driven by negative attitudes toward opposed parties and candidates than by positive attitudes toward one’s preferred party and candidate. Based on an original post-electoral survey of American citizens eligible to vote, we find that about one third of voters cast a negative vote in the 2020 Presidential election.

From a theoretical point of view, we contribute to the existing literature by advancing a composite model for the study of negative voting. Our model combines insights from the classic rational choice literature (i.e., retrospective performance evaluations and rationalization mechanisms) with the most recent contributions from the socio-psychological literature on affective polarization.

Our results provide empirical confirmation for the validity of our composite model. On the one hand, we find that negative voting is linked to both retrospective performance evaluations and strength of partisanship. On the other hand, we highlight the relevance of voters’ affection towards parties and candidates. While despise toward the opposition emerges as a necessary condition for voting “against,” we also find that the tendency towards negative voting is compensated by positive attitudes towards one’s preferred party and candidate. Indeed, what seems to really differentiate positive voters from negative voters does not lie in the extent to which they dislike the opposition—both type of voters are very negative towards the “other” candidate. What makes the difference is whether they like their own candidate enough.

This paper is structured as follows: in the next section, we review the classic literature on negative voting and bridge it with the most recent works on American affective polarization. Then we introduce our original post-election dataset, present our results and a series of robustness tests in turn. We conclude with a discussion of the implications of our results for the emerging debate on negativity and political behavior.

## Negative voting in American Elections

The idea of negative voting is not new in political science. Over a half century ago, V.O. Key (1966, 60) first alluded to the journalistic supposition that “the people only vote against, never for.” And indeed, the conventional wisdom that citizens are largely (as well as increasingly) given to voting against was subjected to empirical scrutiny—with several confirmatory results—by American electoral research since the 1970s. While debunking Key’s coarse claim, this strand of research concluded nonetheless that citizens do not always vote for the candidate they like the most (Gant & Davis, 1984). These studies focused on two types of explanations for negative voting.

Earlier studies on negative voting were largely embedded with the rational choice paradigm, conceiving negative voting as a special case of retrospective voting in elections involving incumbents (Kernell, 1977; Fiorina & Shepsle, 1989). The wear and tear of holding office increases the likelihood of discontent with presidential performance among voters, leading in turn to a higher rate of votes against the incumbent. However, the intuitive value of this empirically testable proposition is counterbalanced by its inability to account for negative votes cast against the challenger, nor about the very existence of negative voting in elections involving no incumbent.

This approach to negative voting is also present in studies at the intersection with political psychology, which largely conceive negative voting as a rationalization mechanism. The motivational cost-orientation hypothesis present in Lau’s (1982, 1985) works conceives it as a strategy privileging cost-avoidance over the maximization of gains. On these bases, subsequent studies drawing from cognitive dissonance theory understood negative voting as the product of the conflicting preferences between party identification and ideology. Negative voting would thus be a strategy to reduce dissonance, by conceptualizing vote choices not as a positive preference for a given party/candidate, but rather as a rejection of the other party/candidate (Gant & Sigelman, 1985;Sigelman and Gant 1989).

This line of reasoning has arguably lost leverage in recent decades due to the profound transformations occurred to the American electorate. A long-term process of partisan dealignment was later followed by a realignment into almost perfectly sorted partisan groups, leading to strong affective polarization across party lines (Dalton, 2018; Levendusky, 2013). Ideological and social sorting have dramatically reduced cross-pressures among the electorate (Mason, 2015, 2018).

A recent strand of scholarship has tackled the electoral consequences of affective partisan polarization through the lens of *negative partisanship*. This literature moves from the social-psychological notion that hostility toward the out-group can develop independently from—and drive support for—the in-group. The existing works on the topic confirm this intuition and highlight an independent relationship between negative partisanship and vote choice even after controlling for positive identifications, both in the United States (Abramowitz & Webster, 2016, 2018; Bankert, 2020) and in comparative perspective (Mayer, 2017; Medeiros & Noël, 2014).

Negative attitudes toward the political out-group concern not only political parties but can also spill over to individual candidates (Bolsen & Thornton, 2021). As the most visible party objects, especially in a Presidential system, candidates are key targets of political aversion. Such contention is supported by recent research on affective polarization, demonstrating that “when people think about the other party, they think primarily about political elites” (Druckman & Levendusky, 2019, 115). The electoral implications of negativity toward candidates are made clear by the finding that “the most important factor in predicting partisan loyalty [in the 2016 American Presidential election] is how an individual feels about the opposing party’s presidential candidate” (Abramowitz & Webster, 2018, 132). It follows that evaluations of (out-party) candidates also act as determinants of the vote, acting alongside positive (in-party) candidate evaluations (Garzia & Ferreira da Silva, 2021b).

We consider all these factors in our explanatory model of negative voting, which features (a) an *instrumental-rational* component, characterized by retrospective performance evaluations and rationalization mechanisms; (b) an *ideological* component grounded on long-lasting political identities; and (c) an *affective* component, motivated by attitudes toward parties and candidates (for a better discussion, see: Garzia & Ferreira da Silva, 2021a: 2).

## Data and Measures

Our analyses rely on a CAWI survey conducted by Qualtrics International. The sample was drawn from an actively managed, double-opt-in market research panel.^1^ It is representative of the American voting population in terms of age, gender, and macro-region of residence (fieldwork dates: November 9–November 29 2020; total *N*=1064).^2^

Despite the limitations associated with online panels, past concerns about data quality vis-à-vis CATI have now been essentially mitigated in relation to empirical research into the determinants of vote choice (Sanders et al., 2007). As a matter of fact, numerous state-of-the-art election studies including ANES nowadays field at least part of their components via online panels. While some analyses still argue that the survey mode produces meaningful differences in accuracy (Chang & Krosnick 2009; Malhotra & Krosnick 2007), others find that Internet, mail-back, and telephone surveys perform similarly (Ansolabehere & Schaffner, 2014; Breton et al., 2017). Ansolabehere and Schaffner (2014: 301) highlight the importance of the recent developments in “constructing, matching, and weighting opt-in Internet panels” over the past decade. In this regard, Qualtrics online recruitment panels have been demonstrated to constitute the most demographically and politically representative solution for the United States: for example, “on partisanship, voter registration, and vote in the 2012 election, Qualtrics is indistinguishable from the 2014 GSS or the 2012 American National Election Studies” (Boas et al., 2020: 240).

Existent research has relied on two alternative measurement strategies of negative voting. Some studies resorted to *indirect* measures, by either recoding open-ended answers (Gant & Davis, 1984), presidential approval scores (Kernell, 1977), or constructing measures of negative evaluations from collapsed feeling thermometers (Maggiotto & Piereson, 1977). Because of its wide availability, the latter option could have the advantage of allowing for a longitudinal assessment of the importance of negative evaluations for voting behavior. However, the distinction between such measures of negative voting and the commonly used strategies to measure negative partisanship, for example, would be very thin. Most importantly, an indirect measure of negative voting based on feeling thermometers would render impossible the consideration of the affective component, tapping into (negative) attitudes toward parties and candidates, since those are measured precisely through the same feeling thermometers used for indirect measures. Moreover, the focus of measurement is on attitudes rather than reported behavior (as shall become clear in the next section, many individuals may hold stronger negative than positive attitudes towards parties/candidates, but still cast a positive vote). For this reason, we deem more appropriate to employ a *direct* measure of negative voting by asking respondents whether they view their vote as more of an expression of support for their preferred candidate or against the opponent (Gant & Sigelman, 1985; Sigelman and Gant 1989). This strategy enables placing negative voting as a dependent variable, which is measured through the question: “Would you say your vote is more a vote for Trump [Biden] or more a vote against Biden [Trump]?“ In the analyses that follow, all respondents deeming their vote as more of a vote *against* are thus considered “negative voters.”

## Results

The share of negative voters in our dataset amounts to 30.2% of all respondents declaring to have cast a vote in the 2020 US Presidential election. Table 1 situates this figure in relation to the few previous studies reporting data on negative voting. Despite the highly polarizing context of the 2020 US Presidential election, and the peculiarities of the candidates involved, the share of negative voting is somewhat in line with earlier elections.

**Table 1.** Shares of negative voters in US Presidential elections.

Year	% Negative Voters	Incumbent
1964	31	Yes
1980	46	Yes
1984	30	Yes
2008	30	No
2016	49	No
2020	30	Yes

*Sources:* For 1964 and 1980: Gant and Sigelman (1985); for 1984: Sigelman and Gant (1989); for 2008 and 2016: Geiger (2016); for 2020: Author’s own elaboration.

Importantly, the proportion of negative voters among Biden supporters (39%) widely exceeds that of negative voters among Trump supporters (18%)—thus, confirming the notion that office holders are the main target of negative voting. As for the distribution of negative voters among incumbent and challenger supporters in previous studies, data is only available for the 1984 election (Sigelman and Gant, 1989): 15% of negative voters among the incumbent’s supporters; 48% of negative voters among the challenger’s supporters. Thus, also in this instance the incumbent was the main target of negative voting.

The theoretical relevance of retrospective considerations for negative voting in the 2020 election is further supported by a bivariate analysis of economic performance assessments. Among negative voters, the proportion of respondents declaring that the American economy has gotten worse/much worse in the last year amounts to 61%. Among positive voters, the same figure amounts to almost a half (33%). Unfortunately, we are unable to compare these figures with previous studies due to data unavailability.

**Figure 1.**
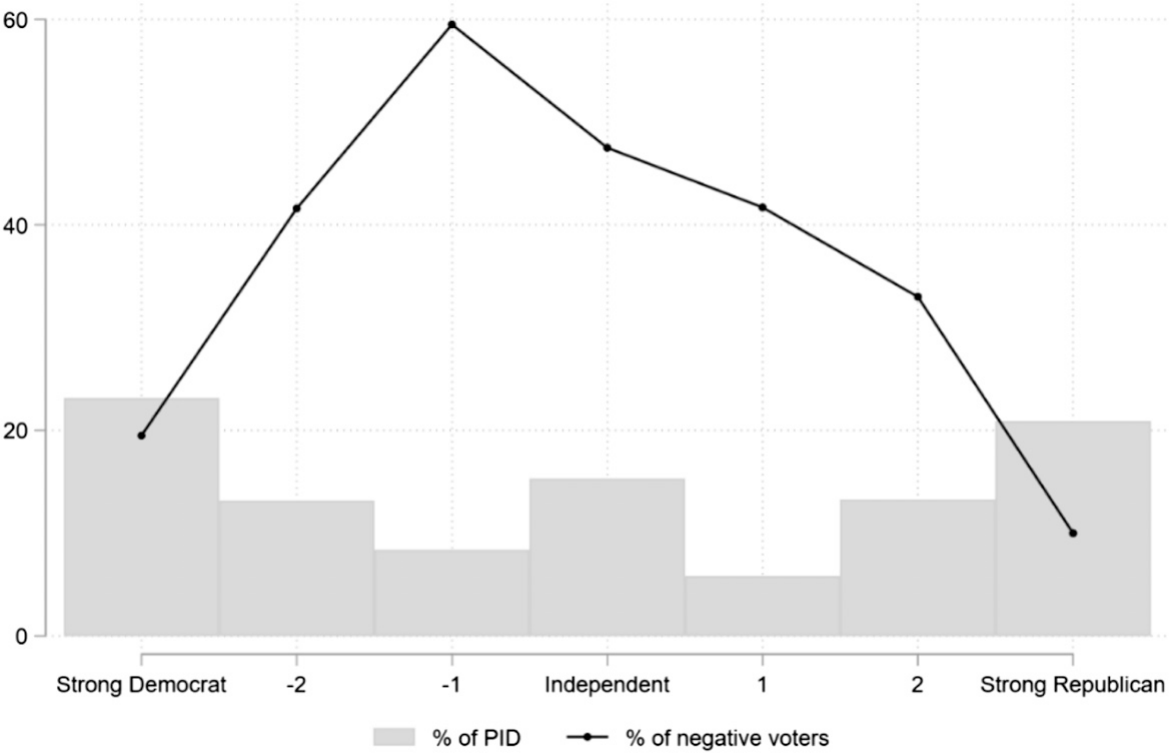
Share of negative voters by party identification (7-point scale).

Based on the findings from this instrumental-rational component alone, one could conclude that negative voting is merely an act of opposition to an out-party incumbent. Indeed, Democratic respondents report higher rates of negative voting than Republican respondents. Yet, it is worth noting that strength of party identification matters for negative voting regardless of its direction (see Figure 1). Among strong partisans, the proportion of negative voters is just 15%, which then goes up to 37% among weak partisans and up to over 50% among leaners/independents.

While it could be expected that long-lasting political identities catalyze negative voting, it appears that they mostly defuse it. Inasmuch as strong partisans may feel aversion toward their opponents, they also feel more positive toward their in-party, on average. Hence, these voters are arguably less likely to cast a vote primarily against a party or candidate. While negative voting is thus at its lowest among strong supporters, it gains traction among voters with weaker partisan ties.

This reasoning is further corroborated by our bivariate assessment of negative voting and party/candidate affect. Table 2 compares the mean scores of in-party/candidate love and out-party/candidate hate for positive and negative voters, respectively. These measures, as developed by Finkel et al. (2020), allow for comparing the strength of in-party/candidate love relative to the neutral point of the feeling thermometer (in-party/candidate score—50) with the strength of out-party/candidate hate (50—out-party/candidate score).

**Table 2.** Mean scores for love and hate thermometer measures, by positive/negative voting.

Year	Positive voters	Negative Voters	t-test
In-party love	27.7 (21.3)	8.5 (25.4)	19.2^***^
Out-party hate	22.5 (30.9)	28.2 (22.7)	**−**5.7^**^
*In-party love–Out-party hate*	*5.2 (36.0)*	*−19.7 (32.7)*	**−24.9** ^***^

In-candidate love	32.9 (19.7)	9.3 (24.3)	23.6^***^
Out-candidate hate	31.0 (27.8)	40.6 (16.2)	**−**9.6^***^
*In-candidate love–Out-candidate hate*	*1.9 (31.4)*	*−31.3 (28.2)*	**−29.4** ^***^

*Note:* Cell entries are mean values of the covariates of interest for positive and negative voters respectively, with standard deviations in parentheses. Significance levels for the t-tests are as follows: ***p < .001 **p < .01.

Positive voters exhibit relatively high levels of in-group love and out-group hate. On average, they “love” their party and candidate more than they “hate” the opponents. To the contrary, negative voters report almost neutral feelings for their chosen party and candidate, but strong negative feelings towards the opponent—thus, well-fitting the very notion of negative voting. However, contrary to the expectation that negative voters would be distinguishable for the extreme negative views of out-group parties and candidates, we find that while they are indeed more negative than positive voters, the key distinctive feature between these two groups lies in their degree of in-party/candidate liking. This is clearly visible through an inspection of the t-test coefficients: the mean difference in the degree of in-party/candidate love among the two groups is indeed much greater than the difference in the degree of out-party/candidate hate. Thus, if both groups have in common a strong disaffect for the out-party/candidate, negative voters are uniquely characterized by lack of affect for their supported party/candidate. It could then be argued that positive voters approximate the ideal-type of affectively polarized voters, in the sense that they affectively situate the two parties in different poles, whereas negative voters seem to resemble more closely the notion of negative partisanship, conceiving the development of negative political identities independent from the existence of positive party/candidate attachments (Abramowitz & McCoy, 2019; Bankert, 2020).

It is also interesting to note that both groups of voters report a higher degree of hate towards opposing candidates than the respective parties. This finding is in line with the intuition that negative candidate evaluations (rather than party evaluations) may exert the strongest role in models of negative voting in US presidential elections (Abramowitz & Webster, 2018: 132).

Table 3 presents the estimates from our logistic regression models of negative voting (full details on the variable coding scheme are available in Supplemental material). The dependent variable is coded “0” for positive voters and “1” for negative voters. Abstainers are thus excluded from the analysis due to the configuration of the dependent variable, yielding a total *N*=823. All models include standard sociodemographic variables (age, gender, macro-region of residence, educational level, employment status, religiousness) as well as ideology, to control for the higher propensity among liberal voters to cast an anti-Trump vote. Descriptive statistics of variables included in the analysis are presented in Supplemenatal material. Aside from age (measured in years), all other variables have been rescaled to range from 0 to 1.

**Table 3.** A multivariate assessment of the attitudinal components of negative voting.

	(1)	(2)	(3)	(4)	(5)
Age	.02 (.01)*	.02 (.01)**	.02 (.01)**	.03 (.01)**	.02 (.01)**
Gender	-.30 (.18)	-.22 (.19)	-.15 (.20)	-.16 (.20)	-.25 (.20)

*Region (baseline: North-East)*					
Midwest	.67 (.26)*	.53 (.27)*	.36 (.29)	.35 (.29)	.40 (.29)
South	.24 (.26)	.18 (.27)	.14 (.28)	.14 (.28)	.21 (.28)
West	.54 (.26)*	.43 (.27)	.24 (.29)	.24 (.29)	.29 (.28)

Education	.57 (.67)	.58 (.67)	.37 (.72)	.40 (.72)	.44 (.72)
Employed	−.29 (.20)	−.34 (.20)	−.44 (.22)*	−.45 (.22)*	−.40 (.22)
White	−.00 (.24)	.01 (.25)	.04 (.27)	.05 (.27)	.03 (.26)
Religious	−.62 (.19)**	−.62 (.20)**	−.58 (.21)**	−.61 (.22)**	−.48 (.21)*
Ideology	−.13 (.35)	−.39 (.37)	−.54 (.39)	−.53 (.39)	−.45 (.39)
					
Retrospective economy	1.99 (.31)***	−1.93 (.33)***	−1.67 (.36)***	−1.67 (.36)***	−1.47 (.34)***
Weak partisan	−.48 (.21)*	−.27 (.22)	−.44 (.24)	−.42 (.25)	−.39 (.24)
Strong partisan	−1.65 (.21)***	−1.10 (−23)***	−1.07 (.24)***	−1.03 (.25)***	−1.26 (.23)***
					
In-party love	—	−2.74 (.42)***	—	.01 (.57)	—
Out-party hate	—	.26 (.40)	—	−.37 (.56)	—

In-candidate love	—	—	−5.14 (.52)***	−5.12 (.62)***	—
Out-candidate hate	—	—	1.99 (.60)**	2.27 (.74)**	—
					
In-party love–Out-party hate	—	—	—	—	1.07 (.81)
In-candidate love–Out-candidate hate	—	—	—	—	−8.83 (1.05)***

Constant	−.14 (.58)	1.33 (.67)*	1.47 (.82)	1.45 (.84)	2.89 (.75)***
*N*	823	823	823	823	823
Pseudo R-squared	.19	.24	.33	.33	.31
AIC	839.99	798.70	710.10	713.68	728.93
BIC	905.97	874.10	785.51	798.51	804.34

*Note:* Table entries are logistic regression coefficients with standard errors in parentheses. *** *p* < .001 ** *p* < .01 * *p* < .05

As suggested by the descriptive analysis, the results indicate that negative assessments of the economy and the absence of partisan ties increase the likelihood of negative voting (Model 1), thus, supporting the relevance of both instrumental-rational considerations and long-standing ideological identifications. In line with the descriptive evidence, it is not strong partisans but independents who are more likely to cast a negative vote. By prompting higher levels of in-group love to counterbalance out-group hate, strong partisanship appears to shield individuals from negative voting. Thus, for strong partisans, there does not seem to be a behavioral manifestation of their negative attitudes toward out-partisans in the form of negative voting. Older and more religious individuals also appear more prone to cast a negative vote.

**Figure 2.**
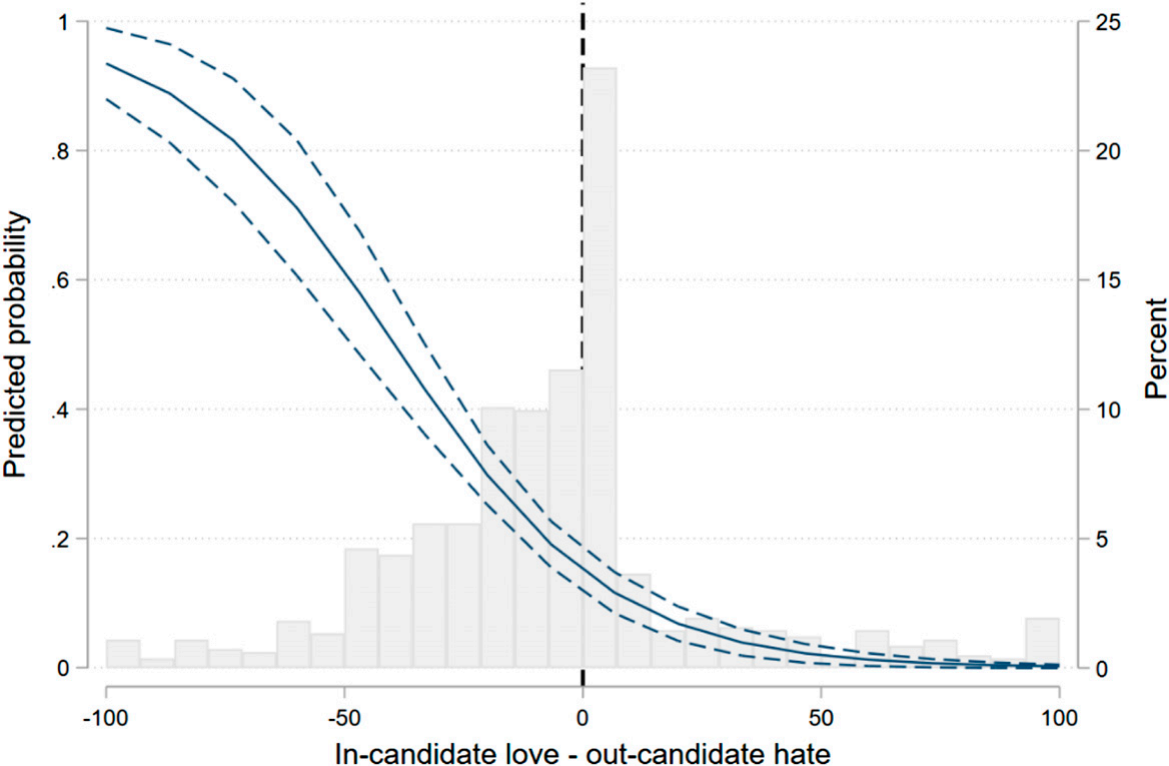
Predicted probabilities of casting a negative vote. *Note:* The histogram reflects the distribution across values of the independent variable.

Models 2 and 3 introduce attitudes towards parties and candidates, respectively. In Model 2, out-party attitudes do not have a significant effect, while (the lack of) in-party love is the strongest predictor of negative voting. In Model 3, the effect of in-candidate love is more than twice the coefficient size of the out-candidate hate variable. These coefficients do not tell us that negative—or positive—voters do not hate; they both hate to a considerable extent. What they convey is that, in line with the descriptive results presented in Table 2, in-group (dis)affect is the crucial factor in explaining the choice to cast a negative voting. The same conclusion emerges when party and candidate attitudes are jointly included in Model 4. However, in this model in-/out-party attitudes become no longer significant, revealing that the role of these affective considerations in negative voting manifests primarily through candidate attitudes. The final model replaces these measures by the respective differentials (Finkel et al., 2020), confirming the preponderance of candidate-based over party-based considerations (Model 5). Recall that above-zero values on these variables reflect greater in-group love than out-group hate, while below-zero values reflect greater out-group hate than in-group love. As suggested by the negative coefficient, the predicted probability of casting a negative vote is substantially higher among individuals for whom out-candidate hate outweighs in-candidate love (see Figure 2).

The predicted probability of casting a negative vote is rather low whenever individuals hold more favorable views of the in-party candidate than unfavorable views of the out-party candidate (positive values). In the modal category of voters loving their own candidate just a little more than they hate the opponent, the estimated probability of casting a negative voting is below 20%. Yet, 59% of individuals have negative values in the differential. For them, the probability of negative voting is quite higher—casting a negative vote quickly becomes more likely than not as values in the original independent variable approach -50.

In sum, the results of the regression analyses provide empirical support for our composite theoretical framework. Retrospective economic assessments, strong partisan identities, and in-group/out-group affect are the most relevant factors accounting for negative voting in the 2020 US Presidential election. These findings highlight both the multicausal nature of this phenomenon, and the changing nature of its determinants.

## Robustness

A split sample re-estimation of Model 5 across Biden and Trump voters yields very similar results (see Supplemental material), specifically concerning the coefficient of the “In-candidate love–out-candidate hate” variable (b_Biden_ = -7.46***; b_Trump_ = -11.10***). The inclusion of an interaction term between this variable and vote choice to test for the difference in effects across the two groups resulted not significant (*p* = .138). The akin results across party supporters suggest that negative voting operates in very similar ways across distinct types of voters.

While we followed previous studies and relied on feeling thermometer scores to measure negative partisanship, it could be argued that such measures suffer from differential item functioning (Osterlind & Everson, 2009). Moreover, as explained by Bankert (2020: 6), measures of negative partisanship drawn from feeling thermometers “are not equivalent to the identity-based conceptualization and measurement of partisanship that has gained popularity in the U.S., making it hard to accurately compare and contrast the effects of negative and positive partisanship.” Feeling thermometers have also been criticized as measures of candidate assessments (Fiorina, 1981). To address these issues, Model 5 was re-estimated using multi-item positive and negative partisan identity scales (Bankert, 2020) and an additive index of four politically relevant personality traits (i.e., honesty, competence, empathy, and leadership). Rescaling the variables, we have mimicked the original in-party/candidate love and out-party/candidate hate measures. The model estimation with these refined measures—which, to the best of our knowledge, were unavailable in simultaneous in previous studies— yields similar results (see Supplemental material). Yet, in this specification, party-based considerations appear to have a small significant effect, possibly explainable by the identity-based measures of (negative) partisanship used instead of feeling thermometers.

## Discussion and Conclusions

Our revisitation of negative voting in American elections provides a new insight into the concept, while speaking to its relevance to understand present-day presidential contests. Contrary to previous analyses of negative voting, which mostly conceive it either as a special case of retrospective voting in elections involving incumbents (Kernell, 1977), or as the product of conflicting ideological and partisan identities (Gant & Sigelman, 1985; Sigelman and Gant 1989), our findings assert the relevance of considering a more complex set of factors. The results from this study show that, parallel to retrospective performance assessments and long-standing political identities, an affective component, motivated by (negative) attitudes toward parties and candidates, is crucial to explain negative voting in the 2020 US Presidential election. Linking negative voting to the climate of political aversion in American society, the analysis reveals that comparative candidate evaluations are the main factor associated with negative voting in the 2020 US Presidential election. The likelihood of negative voting is higher whenever out-candidate hate outweighs in-candidate love, while party-based considerations appear less related—an indication supported by recent studies (Abramowitz & Webster, 2018).

Most importantly, while this study confirms the notion that negative attitudes outweigh positive attitudes for negative voters, our analysis sheds light on the relationships underlying these dynamics and their behavioral translation into patterns of vote choice. More specifically, it shows that positive and negative voters are more similar on the extent to which they dislike out-parties/candidates than on the extent to which they like they own. Therefore, the key different between these groups of voters appears to reside not on the negativity toward the others, as is often assumed, but on the degree of affection toward supported party/candidate. Since this affection is more present among partisans, they are more shielded from negative voting. Along with recent studies, this finding shares the “positive message that strong partisans are not condemned to demonize the other party” (Bankert, 2020: 1), at least when it comes to the point of motivating patterns of voting *against*.

On these bases, the consequences of polarization and growing negativity in American society and politics do not fall exclusively upon partisans. Independents are similarly vulnerable to negativity, yet with the aggravation of this not being counterbalanced by in-group affect—a factor which we have shown to be determinant in reducing negative voting. The higher rates of negative voting among independents resonate with previous accounts portraying them as more cynical than the average voter (Dalton, 2004). Recent scholarship has shown that the rise of extremely negative feelings for presidential candidates represent genuine unappreciation of their profile rather than a mere byproduct of the overreaching process of affective partisan polarization (Christenson & Weisberg, 2019). By this token, the problem may lay in the supply rather than in the demand: mediocre candidates have been increasingly struggling to collect support among their ranks, let alone conquer independents. If parties are unresponsive to their discontent, independents are left with an exit or voice choice: either abstain or vote against. While the 70% of voters in our sample who still are not negative voters may offer some comfort, we remain in the dark as to number of abstainers who choose not to participate because their negative views of parties or candidates outweighs their positive views. The conceptualization of measurement of negativity among abstainers could be a potential research path into understanding how these dynamics affect levels of electoral participation beyond our analyses focused exclusively on voters.

Finally, these findings show that even taking what some consider an extreme case of party polarization and negativity—the 2020 US Presidential election— negative voters remain a minority among the electorate. Moreover, regardless of Trump and the COVID-19 pandemic, levels of negative voting in the 2020 election were not significantly different than in past American Presidential contests (see Table 1). Notwithstanding the increased incentives to cast a vote *against*, reassuringly, for a large majority of voters, the negative attitudes towards their rival parties/candidates did not translate into negative forms of voting behavior. However, the context of this election may have inflated the role of affective considerations over ideological or instrumental-rational in explaining negative voting, compared to previous elections. While negative voting is a lasting feature of Presidential elections, measuring and explaining the changing dynamics of negative voting in US Presidential elections will likely remain as pressing in the future.

So far, negative voting has almost exclusively been studied in the United States (for an exception, see: Kim, 2021). Yet, there are reasons to believe that it could be present in multi-party systems as well. While some unique features of the American case provide stronger incentives for negative voting (two-party system, partisan media, extensive use of negative campaigning, etc.), recent evidence suggests that some of these factors are extending to multi-party democracies as well. Affective polarization and negative campaigning, it is argued, are now a feature of many established parliamentary democracies (Maier & Nai, 2021; Reiljan, 2020; Wagner, 2021). Their diversity in terms of political and institutional configurations could introduce further variance into the patterns of negative voting, justifying a comparative approach beyond the American case.

## Supplemental Material

sj-pdf-1-apr-10.1177_1532673X221074633 – Supplemental Material for The Electoral Consequences of Affective Polarization? Negative Voting in the 2020 US Presidential Election Click here for additional data file. Supplemental Material, sj-pdf-1-apr-10.1177_1532673X221074633 for The Electoral Consequences of Affective Polarization? Negative Voting in the 2020 US Presidential Election by Diego Garzia and Frederico Ferreria da Silva in American Politics Research

## Notes

1. Participants received an email invitation informing them that the survey is for research purposes only and how long the survey is expected to take (8–10 minutes). All participants received a compensation based on the length of the survey, their specific panelist profile, and target acquisition difficulty. The compensation may include cash, airline miles, gift cards, redeemable points, charitable donations, sweepstakes entrance, and vouchers. Qualtrics International Inc. was responsible for the fair compensation of participants in line with relevant wage standards currently operating in the US. No deception was involved in this study.

2. While acknowledging that post-electoral surveys carry problems such as ex-post rationalization, which could affect the reliability of our variables of interest, the alternative option (a pre-electoral survey) would be, if anything, even more problematic. Extrapolating vote choice from pre-electoral vote intentions would lead to inaccuracy, since they tend to be more volatile. Moreover, drawing from vote intentions would exclude late deciders, a significant part of the voting population. Most importantly, early voting, mail voting, and the specifics of the 2020 US election would render extremely difficult to exclude individuals who had already cast a vote, even if the survey had been run previous to the election. To fully isolate the survey from individuals who had already cast a vote, we would need to have fielded the survey too early in the campaign, thus, capturing vote intentions completely insulated from the massive campaign effects observable in American Presidential elections, again increasing inaccuracy.
