# Supplementary material for: The Electoral Consequences of Affective Polarization? Negative Voting in the 2020 US Presidential Election
Source: Am Polit Res. 2022 Jan 27;50(3):303–11. doi: 10.1177/1532673X221074633 (PMC9028101; doi:10.1177/1532673X221074633)

ONLINE APPENDIX A

**Variable**	**Coding scheme**
Negative Vote	0. Cast a vote more for a candidate; 1. Cast a vote more against a candidate
Age	*Numeric*
Gender	0. Male; 1. Female
Region: Northeast	0. No; 1.Yes
Region: Midwest	0. No; 1.Yes
Region: South	0. No; 1.Yes
Region: West	0. No; 1.Yes
Education	
Employed	0. Not currently employed; 1. Currently employed
White	0. Not white; 1. White
Religious	0. Not religious; 1. Religious
Ideology	1. Extremely liberal; 2. Liberal; 3. Slightly liberal; 4. Moderate, middle of the road; 5. Slightly conservative; 6. Conservative; 7. Extremely conservative
Economy	1. Gotten much worse; 2. Gotten worse; 3. Stayed about the same; 4. Gotten better; 5. Gotten much better
Party identification	-3. Strong Democrat; -2. Weak Democrat; -1. Independent Democratic; 0. Independent; 1. Independent Republican; 2. Weak Republican; 3. Strong Republican
In-party love	0. Minimum in-party love; 100. Maximum in-party love
Out-party hate	0. Minimum out-party hate; 100. Maximum out-party hate
In-party love – Out-party hate	-100. Minimum in-party love/ Maximum out-party hate; 100. Maximum in-party love/Minimum out-party hate
In-candidate love	0. Minimum in-candidate love; 100. Maximum in-candidate love
Out-candidate hate	0. Minimum out-candidate hate; 100. Maximum out-candidate hate
In-candidate love – Out-candidate hate	-100. Minimum in-candidate love/ Maximum out-candidate hate; 100. Maximum in-candidate love/Minimum out-candidate hate
Trait index	1. Not well at all; 2. Slightly well; 3. Moderately well; 4. Very well; 5. Extremely well

ONLINE APPENDIX B

Descriptive statistics for variables included in the regression analysis

Variable	N	Mean	St. Dev.	Min	Max
Vote: Biden	870	0,59	0,49	0	1
Vote: Trump	870	0,41	0,49	0	1
Negative Vote	870	0,30	0,46	0	1
Age	1064	46,31	17,66	18	88
Gender	1064	0,50	0,50	0	1
Region: Northeast	1064	0,22	0,42	0	1
Region: Midwest	1064	0,25	0,43	0	1
Region: South	1064	0,27	0,44	0	1
Region: West	1064	0,26	0,44	0	1
Education	1064	11,82	2,50	2	17
Employed	1064	0,48	0,50	0	1
White	1064	0,77	0,42	0	1
Religious	1064	0,39	0,49	0	1
Ideology	1015	-0,03	1,78	-3	3
Economy	1029	2,87	1,30	1	5
Party identification	980	-0,09	2,27	-3	3
Weak partisan	980	0,26	0,44	0	1
Strong partisan	980	0,44	0,50	0	1
In-party love	870	21,91	24,24	-50	50
Out-party hate	870	24,24	28,80	-50	50
In-party love – Out-party hate	870	-2,32	36,79	-100	100
In-candidate love	870	25,78	23,82	-50	50
Out-candidate hate	870	33,88	25,27	-50	50
In-candidate love – Out-candidate hate	870	-8,10	34,10	-100	100
PPID (In-party)	660	2,29	0,80	1	4
NPID (Out-party)	761	2,17	0,91	1	4
PPID – NPID (In-party – Out-party)	660	0,09	0,84	-2,13	3
Trait index (In-candidate)	855	3,86	1,02	1	5
Trait index (Out-candidate)	857	1,75	1,11	1	5
Trait index (In-candidate – Out-candidate)	850	2,10	1,56	-3	4

ONLINE APPENDIX C

The effect of candidates' love-hate differential by candidate choice

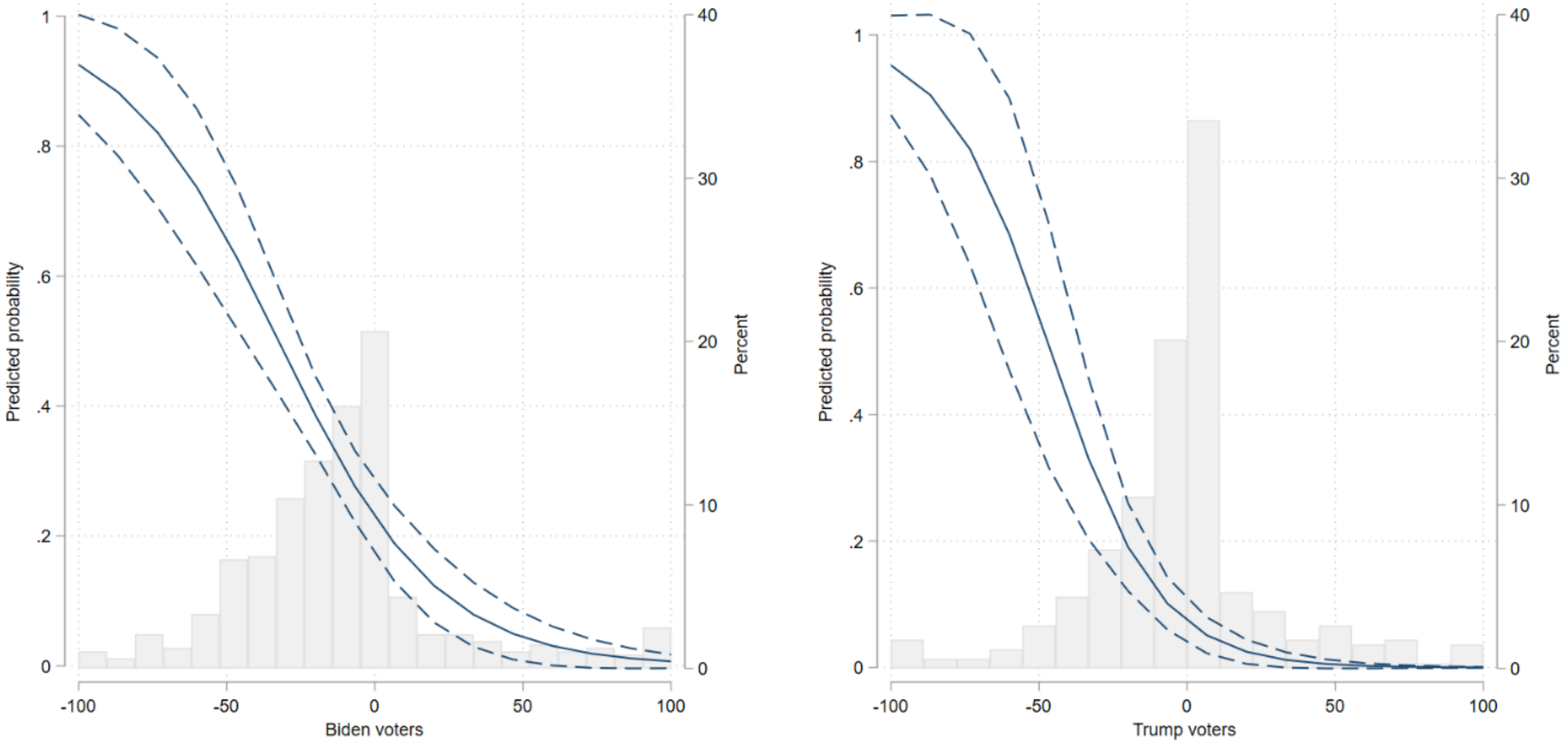

ONLINE APPENDIX D

PPID, NPID and candidate trait assessments as predictors of negative voting

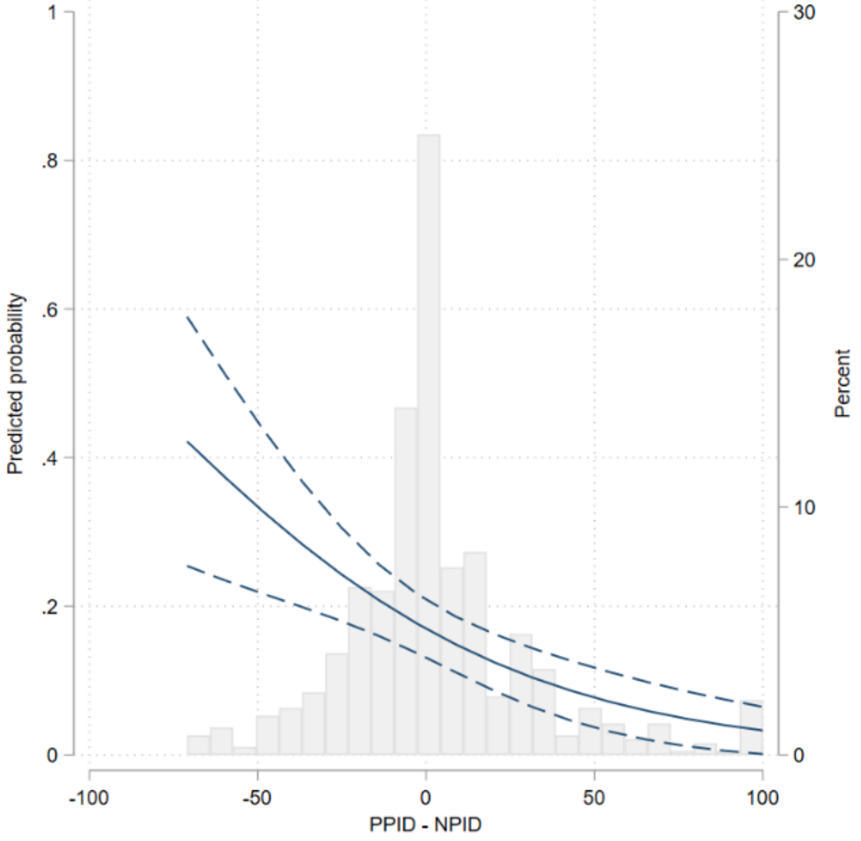

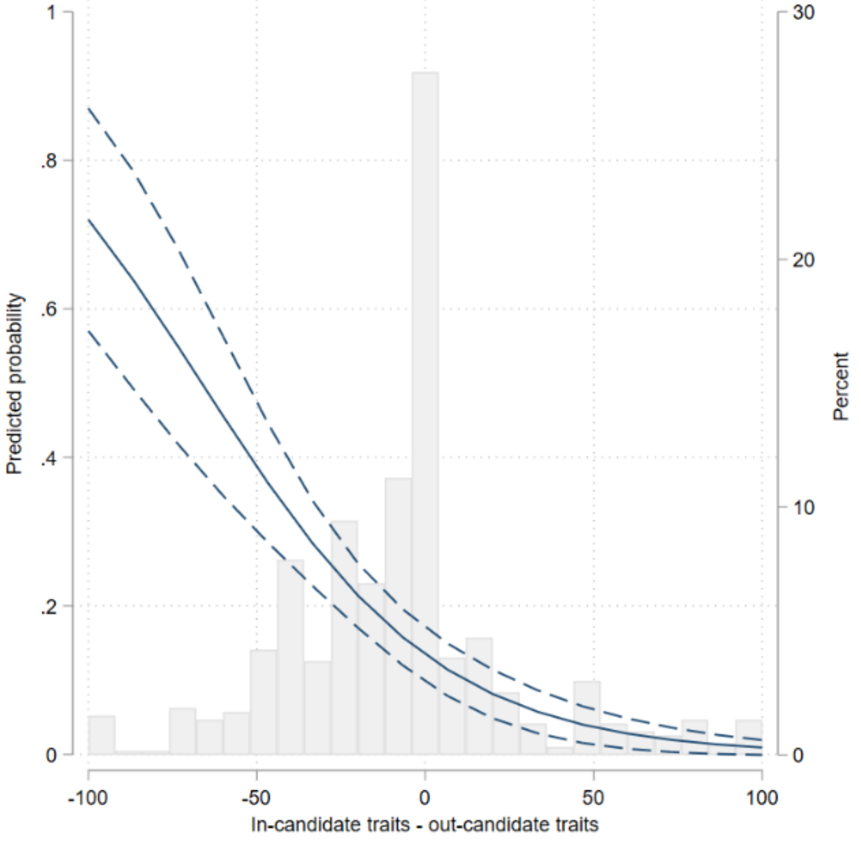

Supplement: sj-pdf-1-apr-10.1177_1532673X221074633 – Supplemental Material for The Electoral Consequences of Affective Polarization? Negative Voting in the 2020 US Presidential Election [file sj-pdf-1-apr-10.1177_1532673X221074633.pdf]
